# Micro-planning in a wide age range measles rubella (MR) campaign using mobile phone app, a case of Kenya, 2016

**DOI:** 10.11604/pamj.supp.2017.27.3.11939

**Published:** 2017-06-22

**Authors:** Amina Ismail, Collins Tabu, Iheoma Onuekwusi, Samuel Kevin Otieno, Peter Ademba, Peter Kamau, Beatrice Koki, Anthony Ngatia, Anthony Wainaina, Robert Davis

**Affiliations:** 1 American Red Cross, US; 2 Ministry of Health, EPI program; 3 World Health Organization, Kenya Country Office, EPI; 4 Kenya Red Cross; 5 Clinton Health Access Initiative; 6 Center for Public Health Diagnosis

**Keywords:** Micro plan, phone app, doforms, cost, county, sub county, wards, management

## Abstract

**Introduction:**

A Measles rubella campaign that targeted 9 months to 14 year old children was conducted in all the 47 counties in Kenya between 16th and 24th of May 2016. Micro-planning using an android phone-based app was undertaken to map out the target population and logistics in all the counties 4 weeks to the campaign implementation instead of 6 months as per the WHO recommendation. The outcomes of the micro-planning exercise were a detailed micro-plan that served as a guide in ensuring that every eligible individual in the population was vaccinated with potent vaccine. A national Trainer of Trainers training was done to equip key officers with new knowledge and skills in developing micro-plans at all levels. The micro planning was done using a mobile phone app, the doforms that enabled data to be transmitted real time to the national level. The objective of the study was to establish whether use of mobile phone app would contribute to quality of sub national micro plans that can be used for national level planning and implementation of the campaign.

**Methods:**

There were 9 data collection forms but only forms 1-7 were to be uploaded onto the app. Forms 8A and 9A were to be filled but were to remain at the implementation level for use intra campaign. The forms were coded; Form 1A&B, 2A, 3A, 4A, 5A, 6A, 7A, 8A and 9A The Village form (form 1A&B) captured information by household which included village names, name of head of household, cell phone contact of head of household, number of children aged 9 months to 14years in the household, possible barriers to reaching the children, appropriate vaccination strategy based on barriers identified and estimated or proposed number of teams and type. This was the main form and from this every other form picked the population figures to estimate other supplies and logistics. On advocacy, communication and social mobilization the information collected included mobile network coverage, public amenities such as churches, mosques and key partners at the local level. On human resource and cold chain supplies the information collected included number of health facilities by type, number of health workers by cadre in facilities within the village, number of vaccine carriers and icepacks by size, refrigerators and freezers. All these forms were to be uploaded onto the phone app. except form 8A, the individual team plan, which was to be used during implementation at the local level. Android phone application, doforms, was used to capture data. Training on micro planning, data entry and doforms app was conducted at National, County, Sub-county and ward levels using standardized guidelines. An interactive case study was used in all the trainings to facilitate understanding. The App was also available on Laptops through its provided web-application. The app allowed multiple users to log in concurrently. Feedback on all the variables were obtained from the team at the Ward level. The ward level team included education officers or teachers, village elders, community health workers and other community stakeholders. Only the Ward level was allowed to collect information on paper and that information was subsequently transferred to the phone-based app, doforms, by health information officers. The national, county and sub county were able to access their data from the app using a password provided by the administrator.

**Results:**

Real time data was received from 46 of 47 counties. One county (Marsabit) did not participate in the micro plan process. Over 97% (283/290) of the sub counties responded and shared various information via the app. Different data forms had different completion rates. There was 100% completion rate for the data on villages and target population. Much valuable information was shared but there was no time for the national and county level to interrogate and harmonize for proper implementation. The information captured during the campaign can be used for routine immunization and other community based interventions. Electronic data collection not only provided the number of children but provided the locations also where these children could be found.

**Conclusion:**

Despite the limitations of time to harmonize the micro plans with the national plan, the micro planning process was a great success with 46/47 counties responding through the mobile phone app. Not only did it provide the numbers of the target children, it further provided the places where these children could be found. There was timely data transfer, data integrity, tracking, real time data visualization reporting and analysis. The app enabled real time feedback to national focal point by data entry clerks as well as enabling trouble shooting by the administrator. This ensured campaign planning was done from the lowest level to the national level.

## Introduction

The Microplanning was done 4 weeks before the campaign implementation instead of 6 months as per the WHO recommendation. Micro planning data collection forms were developed based on forms used in Kenya’s 2002 measles catch up campaign and on WHO generic guidelines [1]. A list of all 1450 wards in Kenya was obtained from Independent Electoral and Boundaries Commission [2]. A global evaluation on progress towards measles elimination 2000-2015 showed that none of the milestones were met [3]. Global MDG 4 had measles vaccination coverage as an indicator to measure progress. The last Kenya population and housing census was done in 2009 and reported a total population of 38,610,097 [4]. The annual population growth rate is estimated at 3.0%, live births 3.6%, surviving infants 3.4%. The 2015 population is projected to be 44,758,100 of which 1,521,776 is the number of surviving infants and 18,972,922 is estimated to be the population aged between 9 months and 14 years [4]. The antigens currently offered in Kenya routine immunization schedule include BCG, OPV, Measles, DTP-HepB-Hib, PCV10, Rotavirus and Yellow Fever (in endemic sub-counties). Measles vaccination campaigns targeting children aged 9-59 months are conducted every three years in Kenya. Prior to the 2016 catch-up campaign, measles campaigns were conducted in 2002, 2005, 2009 and 2012. In all these campaigns, monovalent measles vaccine was administered. The 2016 campaign offered MR (Measles- Rubella) vaccine. National micro plans have been done before in 2002 and 2013. In both cases, the MOH used hard copy forms. In 2013 the micro plan was able to map all the districts but the information took over one year to be availed at the national level. In 2013, there were also gaps such as some forms being blank and only the budget form got completed as required.

## Methods

### Design of the data forms and data collection process

The manual hard copy forms were coded and uploaded into the mobile software application. The mobile form design utilized basic and advanced design algorithms to minimize time to fill the mobile questionnaire such as automatic calculation of summary data from input data. Location features and stringent data validation mechanisms were put in place to ensure data integrity. The primary use of the location services was to ensure data entered was actually from the intended county with no manipulation whatsoever.

Pretests to ensure form functionality were performed and adjustments made before the questionnaires were given to the end users. Each County was assigned a unique username and password. The latter was the phone number for the County Health Information Officer which facilitated accountability for data entry and accuracy as well as created a link between the Group Administrator and the county teams for troubleshooting.

The forms were labelled similar to the hard copy. Of the nine forms on hard copy, only the individual plans and map were not uploaded on the mobile app. as they were operational tools to be used at the implementation level. For the remaining seven forms, it was compulsory for responses to be filled in tandem with the numbering. One could not fill form 6A before filling out form 1A1B. This brought in the factor of form relationship and dependency as a measure to ensure that no form was skipped and data flow was smooth. Each form depended on the previous form; thus the need to fill the forms consecutively after each other. The relationship added to the data validation and integrity.

### The micro planning process

Micro planning data collection forms were developed based on forms used in Kenya’s 2002 measles catch up campaign and on WHO generic guidelines [1]. A phone application, doforms was used to capture data. Cascade training on MR campaign microplanning and mobile phone use was conducted starting with National TOTs who trained the County teams. County trainers then oriented the Sub County teams who in turn facilitated the Ward teams. Focal points at all levels facilitated the process. There were 9 data collection forms labelled 1A&B, 2A, 3A, 4A, 5A, 6A, 7A, 8A and 9A, but only forms 1-7 were to be uploaded onto the mobile phone app. The Village form (form 1A&B) captured information by household included villages name, head of household, cell phone contact and number of target population in the household, mobile network coverage, public amenities such as churches, mosques, number of health facilities by type, number of health workers by cadre in facilities within the village, number of vaccine carriers and icepacks by size, refrigerators and freezers. Each form captured specific type of information all by village. Form 8A was an individual team movement plan form which was to be filled by the district team to cover the population captured by sheet 1A&B. Form 9A was a Ward map showing major physical features to assist in team distribution. All the Information from all the seven forms were to be uploaded onto the phone app. A list of all 1450 wards in Kenya was obtained from Independent Electoral and Boundaries Commission [2]. Training on micro planning, data entry and doforms app was conducted at National, County, Sub-county and Ward levels using standardized training guidelines. An interactive case study was used in all the trainings to facilitate understanding. The App was also available on Laptops through its provided Web-Application. The app allowed multiple users to log in concurrently. Feedback on all the variables were obtained from the team at the Ward level. The Ward team included staff from the largest health facility in the Ward, key stakeholders and partners at that level such as the village elders, sub chiefs, headmen and community health workers and the Ward political representative. Only the Ward level was allowed to collect information on paper and that information was subsequently transferred to the phone-based app, doforms, by sub county health information officers. The national, county and sub county levels were able to access their data from the app using a password provided by the administrator. Sub County health information clerks transferred the information from hard copies into the relevant format using the mobile phone app., the doforms. Data entry was real time such that the County and National teams received the information as it was entered on the phones by the data clerks.

A central server database was established and used to aggregate the collected data from all the 47 counties. The National and County teams had no influence over the system or data which were monitored by one independent group administrator. For data changes to be effected, the request would be initiated by the data entry person to the group administrator. He would then initiate a dispatch feature to the specific data person who would then return the form with the correct entries to the system. On the first page of the micro plan forms, counties were required to enter the 9 months-14 years proportion of their population which was to be adopted by the respective sub counties. This proportion would then reflect on respective forms where population figures were required. Automatic user login trails was performed by the system at regular intervals which boosted the data security and protected the system from manipulation.

### Results

The microplanning process was done within a very short time, 4 weeks, compared to the 2013 polio micro plans which took more than one year to be submitted to the national level. Three weeks of intensive data entry using the mobile app, had 46/ 47 counties in the country upload data. The mobile phone app was real-time and provided information that could be viewed at different levels. Administrative rights to view data were given to the leadership at both National and County levels. Once the data was uploaded, no edits were allowed at any level. This ensured data integrity and removed the aspect of higher levels tampering with the Ward data. However the hard copies remained at the district level to be used during campaign implementation. Since the forms were related and data could only be filled on the forms sequentially, it was not possible to get missing information in some forms as we had in 2013. Feedback and support was prompt and the records and information officers at sub county level provided the necessary IT support to the sub counties. The app was able to provide GIS position of the person entering the data to stop any one not from the county entering the data. This way we increased ownership of the information by counties and sub counties.

However, despite the success, we also had challenges. The uploading of the data by the sub county teams were not getting uploaded as fast as planned. The app was designed in such a way that one could save an incomplete form as a draft and could retrieve the same form to complete later. The completed forms could then be saved as final and could only be viewed but not changed. We had a challenge where most people had incomplete drafts in the system leading to clogging. This was a bit frustrating for those who wanted to upload completed forms. Only the national administrator and the one entering the forms on the phone at the district level could access the draft forms. The other challenge was that the overall app administrator was an IT expert and had no health background. In some instances the questions from the field were technical. We also had only one person, the administrator who was able to troubleshoot. He could receive many calls from the field asking the same question and this was exhausting. There was also a misconception that since the sub counties had uploaded the data, they did not require the hard copies. As mentioned earlier, not all pages were relevant for national level, such as form 8A which captured the individual team plans. Despite having the hard copies of the micro plans within the districts, the individual team plans form were not used to distribute and monitor vaccination teams and allocate vaccines and other logistics

The data that was collected by the district teams had anomalies on exaggeration of population figures. Most counties had declined the population proportions based on the Kenya Bureau of Statistics figures as provided by the National Vaccines and Immunization Program (NVIP). This led to the number of children aged 9m-14 years shared in the micro plans being substantially higher than that which was projected in official government census documents. Eventually, due to this anomaly, counties ended up using denominators provided by the national level for administrative coverage instead of their micro plans. The populations in the micro plans were inflated for reasons such as budget as well as higher allocation of other resources. The funding from national level was based on the national plan and not the micro plans. This led the counties to abandon the micro plans. The 2 forms 8A and 9A which were to be used during implementation were seldom used.

The mobile app was able to record 53,277 villages in the 46 counties. 4 counties (Muranga, Nandi, Transnzoia and Turkana), had abnormal population figures that were considered as outliers. With the exception of these 4 counties, the micro plans captured more than 3 million children who were not in the national plan. 98% (46/47) had mapped all the places where the target age children could be found. These places included villages, schools, place of work and market place (daily and cyclical). On creation of awareness and communication, mapped churches and mosques, mobile phone networks by village, radio station coverage and extra source of resources in addition to national level resources. 11% of the 46 counties (5/46) did not respond to the questions on cold chain facilities in the sub counties.

**Figure 1:**
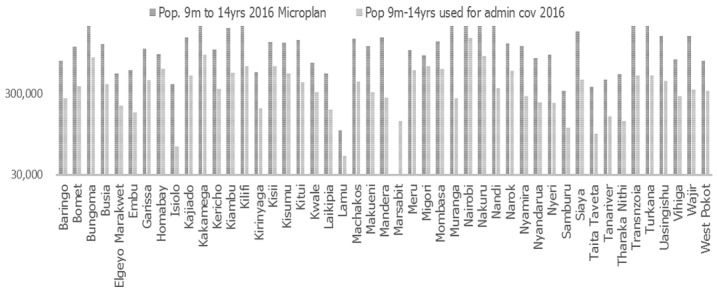
Reported target population in micro plans in comparison to the denominators used in the MR SIAs

The administrative coverage was based on the national denominators and not the micro plan denominators. The micro plan denominators were way higher than the denominators provided for by the national level in all the 46 counties as shown in the Figure 1 below. If the populations captured by the micro-plans were to be used, the average national administrative coverage would have been around 40%.

## Discussion

For the first time after the 2002 measles catch-up campaign, there were data flowing from bottom-up as opposed to the traditional top-bottom. Although the microplanning data was not used in the campaign, the information collected is valuable when planning for routine health activities such as immunization or integrated outreach services. In West Pokot County where routine immunization is low, village population can be used to map strategic interventions. According to the National Vaccination and Immunization Program (NVIP) routine immunization data for 2015, Mandera county, a county in the extreme northern part of Kenya with nomadic population, with several reported measles outbreaks, had a coverage of 29% for MCV1 (MOH,DVI 2015). From their micro plan some villages had populations as high as 70,000. The national statistics bureau estimates a village in Kenya to have an average of 5000 people. Such disparities in population can lead to under estimation of the target population for Mandera. This is the reason why micro plans are important since such details could not be established otherwise. Harmonization between the national and county teams would help address such issues and ensure all children are reached with vaccines. Unfortunately, harmonization between counties and national level was not done due to time constraint.

### Factors to consider while implementing an electronic mobile collection

While on a hard copy, the implementation of skip logic and conditional questions is tedious, the mobile app ensured ease of calculation, automation, reducing errors and time taken to perform entries. There were salient features that made the adoption of the system successful. These included; offline saving capability where the mobile system was capable of capturing data even when offline and this ensured that the team would work flexibly both online and offline. It also had draft retrieval capability where all incomplete forms were saved under the folder ‘drafts’ which would be accessed later for editing before final submission. The automated calculations were in built in the system. The system performed all the calculations and this saved the user from long and tedious calculations which was required in most of the forms. The do-forms app was compatible with all android phones with a base version of 4.2.2 upwards. Most android phones had base version of 4.2. Finally the app had location services. It was able to locate the exact point of data collection to avoid off site data collection. Other features included ability to show the data collection teams that had low batteries or had their devices switched off, all these could be monitored by the administrator who was in Nairobi.

#### Mobility

The process of collecting data was flexible and friendly to the end user. Data was collected even though no computer was available. To enable flexible data collection, the device needed process-driven data collection using smart mobile devices which were portable Further, it did not distract the participating actors in communicating and interacting with each other.

#### Multi-user support

Since different users interacted with the mobile questionnaire, multi-user support was crucial. It was possible to distinguish between different user roles (for example interviewers and subjects) involved in the processing of the electronic questionnaire.

**Support of different questionnaire modes:** generally, two modes; interview and self-rating mode, were used. These two modes of questioning depended on the way the questions were posed, the possible answers that may be given, the order in which the questions are answered, and the additional features provided (e.g. free-text notes).

**Challenges electronic data collection system:** while there exists more benefits to using the electronic method of data collection, there were challenges. During the micro-plan process, internet network connection was a major challenge. A proper audit of network access in the marginalized areas need to be factored in. Availability of a good and stable network infrastructure will ensure the real-time update of data as they flow from the different centers. We also had a challenge on instructions on data entry. We experienced jam in data flow when some counties submitted drafts that were not complete onto to the app. system and delayed to either retrieve and finalize or discard. Entering data in clusters instead of individually onto to the app. was also posed as a challenge as such could not be visualized by the administrator and the person entering the data.

## Conclusion

Despite the limitations of time to harmonize the micro plans with the national plan, the micro planning process was a great success with 46/47 counties responding through the mobile phone app. Not only did it provide the numbers of the target children, it further provided the places where these children could be found. There was timely data transfer, data integrity, tracking, real time data visualization reporting and analysis. The app enabled real time feedback to national focal point by data entry clerks as well as enabling trouble shooting by the administrator. This ensured campaign planning was done from the lowest level to the national level. There is need to develop a frequently asked question page (FAQ) on the phone app. to avoid many phone calls as well as reduce cost of calling. During the training on the mobile phone app, there is need to emphasize on what information is required at the national level and what is required at sub national level. Much of the information was required for implementation at sub national level. What the national level required was mainly the population figures and the advocacy and communication channels. The useful information captured for the campaign can be used for routine immunization as well. The impact of electronic data collection system cannot be over emphasized though there is need to have credible source of population information that can be agreed upon at national and sub national levels. Automation of the micro planning using the mobile app reduced time for data collection. The information collected is useful for routine immunization and other primary health care activities linking to communities.

### What is known about this topic

- Although cellphones and other new technologies are increasingly used in research and health care, very limited data are available to determine their impact. As mobile phone technology approaches 100% reach globally, there are concerted efforts to use the technology to improve health; A systematic literature review (Kati Annelo Kannisto, 2014), shows improved health outcomes due to sms use in different settings;
- Mobile data collection significantly reduces the time for data collection and processing; In Tanzania, a study on ‘’use of electronic protocols to improve provider and client adherence’’, clinicians were trained on using a step by step flowchart in IMCI using a mobile app instead of the traditional paper form: the study established that less time was spent in diagnosis, management and improved outcome of sick children when using the mobile app as compared to the paper form (Mitchell, Marc D., 2012);
- A study on ‘The Mobile Solutions for Immunization (M-SIMU) Trial in Kenya’ showed that mothers brought in their children for vaccination on receiving text messages on their phones (Gibson DG, 2017).

### What this study adds

- This study underscores the efficiency of mobile data in decision making for large vaccination campaigns. Efficiency and accuracy of data heavily relies on the execution of proper controls and use of a reliable system for the given field;
- An automated data collection system is cost effective as opposed to a manual system;
- A bottom-Up approach in data mining in a health field creates a sense of ownership and improves accuracy; a robust micro plan is critical to ensuring a proper and updated data to be used for planning and mobilizing adequate resources.

## Competing interests

The authors declare no competing interest.
